# Representational change and strategy use in children's number line estimation during the first years of primary school

**DOI:** 10.1186/1744-9081-8-1

**Published:** 2012-01-04

**Authors:** Sonia LJ White, Dénes Szűcs

**Affiliations:** 1 University of Cambridge, Department of Experimental Psychology, Centre for Neuroscience in Education, Downing Site, CB2 3EB, UK; 2 Queensland University of Technology, School of Early Childhood, Faculty of Education, Victoria Park Road, Kelvin Grove, 4059, Australia

## Abstract

**Background:**

The objective of this study was to scrutinize number line estimation behaviors displayed by children in mathematics classrooms during the first three years of schooling. We extend existing research by not only mapping potential logarithmic-linear shifts but also provide a new perspective by studying in detail the estimation strategies of individual target digits within a number range familiar to children.

**Methods:**

Typically developing children (n = 67) from Years 1-3 completed a number-to-position numerical estimation task (0-20 number line). Estimation behaviors were first analyzed via logarithmic and linear regression modeling. Subsequently, using an analysis of variance we compared the estimation accuracy of each digit, thus identifying target digits that were estimated with the assistance of arithmetic strategy.

**Results:**

Our results further confirm a developmental logarithmic-linear shift when utilizing regression modeling; however, uniquely we have identified that children employ variable strategies when completing numerical estimation, with levels of strategy advancing with development.

**Conclusion:**

In terms of the existing cognitive research, this strategy factor highlights the limitations of any regression modeling approach, or alternatively, it could underpin the developmental time course of the logarithmic-linear shift. Future studies need to systematically investigate this relationship and also consider the implications for educational practice.

## Background

Estimation is a required skill for everyday life. Numerical estimation skills are an example of what Piaget [1] described as logico-mathematical knowledge. While Piaget did not carry out numerical estimation tasks specifically he considered logic-mathematical knowledge to be the mental relationships between and among objects/representations. Understanding the development of numerical estimation is particularly important to psychologists and educators, as several studies indicate the benefits of advanced estimation skills. For example, many studies (e.g. [2-5]) have determined a strong, positive correlation between the accuracy of numerical estimation and standardized tests of mathematics achievement. Furthermore, LeFevre, Greenham and Waheed [6] propose the tendency of skilful estimators to have a better conceptual understanding of mathematics, as well as better counting and arithmetic skills. Here we provide an investigation of numerical estimation skills at the beginning of primary school. We used a number range familiar to the children and analyzed dependent variables for each target digit in depth. This approach goes beyond studying a potential logarithmic-linear representational shift in estimation and allows further insight into the development of children's estimation strategies.

Several studies (e.g. [2,5,7-11]) have investigated developmental changes in numerical estimation in school-aged children. Estimation requires the translation between alternative quantitative representations. For example presenting a child with a number and asking them to position it on a number line can be described as a translation from a numerical to spatial representation [5].

Much of the research into numerical estimation (hereafter: estimation) has focused on how magnitudes might be mentally represented and how this representation changes with maturity. It is assumed that estimation is based on internal models of magnitudes. Two models attempt to describe the internal representation of number, namely the accumulator (linear) model [12] and the logarithmic model [13]. The accumulator model suggests that magnitudes are represented linearly and that the accuracy of this mental representation decreases with increasing magnitude [8]. The variability of estimations in relation to the magnitudes estimated remains in a constant ratio; this is termed 'scalar variability' [14]. Dehaene [13] argued that quantities are represented in a logarithmic fashion. This mental representation results in an exaggeration of the distance between small number magnitudes in comparison to distances between large number magnitudes. In relation to the core systems of number; namely the *small number system *for small number enumeration and the *approximate number system *(ANS) for larger numerosities [15], it is the approximate number system that would encode the numerosities in an estimation task. Specifically, Halberda and Feigenson [16] found that ANS acuity was still developing in children aged 3-6 years and speculated that sharpening of the ANS was not complete until late in adolescence. Furthermore, Berteletti et al. [7] argues for an approximate number system that is a logarithmic representation first, with numerate children and adults acquiring greater precision, and thus a linear representation. This shift to a linear representation is evident first with familiar number contexts and then subsequently with less familiar number ranges [17].

Many of the developmental studies have used pure numerical estimation with large number scales (e.g. 0-100 and 0-1000: [2,5,10]). On a 0-100 number line, this research ([2,5,10]) purports that both representations are evident and pinpoints a logarithmic-linear shift at around Grade 2 (7-8 years). Booth and Siegler [2] declare a linear best fit for 74% of Grade 2 children in their study; with the remainder of participant behaviors being best represented by a logarithmic model or in a minority of cases, an exponential model. With younger participants the logarithmic-linear distinction is less clear; for example in Siegler and Booth [5], 5% of kindergarteners produced a series of estimates better characterized by the linear than the logarithmic model and 45% were best modeled by a logarithmic representation.

It could also be argued that these results, particularly those of children in kindergarten, could be influenced by the unfamiliarity of the number ranges. Verifying this proposition Ebersbach et al. [8], in a similar 0-100 task, found that only 17% of kindergarteners and 38% of Grade 1 children who participated in the study could count to 100. While this might question the validity of the logarithmic-linear claim, Berteletti et al. [7] utilized 1-10, 1-20 and 0-100 number lines in an estimation task and found evidence for the logarithmic to linear representational shift. Of the preschool aged children (3.5-6.5 years) who participated in the 0-100 task all groups displayed a logarithmic dominant representation. For the 1-10 task the youngest group (approximately 4 years) was best fit by both models, the middle and older groups (approximately 5 and 6 years respectively) were now demonstrating a linear preference for this reduced number scale. In the 1-20 estimation task the youngest group was best fit by a logarithmic model, whereas the middle and oldest groups were equally well represented by logarithmic and linear models. These findings [2,5,7,10] reinforce the belief that child estimation behaviors demonstrates a logarithmic phase prior to linearity and that this transition is evident first with familiar and then unfamiliar number contexts.

Berteletti et al. [7] acknowledges that the exact path that leads from logarithmic to linear representations is still unclear. Siegler, Thompson and Opfer [18] argue it to be a process important to education. Thompson and Siegler [11] interpret the flexibility/variability of behaviors within Siegler's [19] overlapping waves theory; whereby representations and strategies are used selectively when most effective and that individual choice of learned (external) mechanisms contribute to numerical representations. In various adult studies of numerical processing (e.g. [20-23]), it has been determined that many different strategies can be used to solve a single problem, whether that be estimation, multiplication or equation solving. Dowker [20] in her study of expert mathematicians found that individual strategy selection for the same problem can vary between trials. Smith [24], in a study with school aged children and adolescents, investigated reasoning with rational numbers (expressed as fractions) and found that higher level competence required rich and diverse knowledge, numerically specific and invented strategies, as well as those general strategies learned through educational instruction. Huntley-Fenner [25] attributes the variability children demonstrate in estimation tasks as being the result of reduced knowledge of estimation strategies. While the influence of individual strategies was mentioned in the numerical estimation child studies of Siegler and Booth [5] and Siegler and Opfer [10], Barth and Paladino [26] and Ebersbach et al. [8] discussed the use of estimation strategies and proposed the need for an alternative modeling approach in order to capture child behaviors. The alternative models were found to factor in the use of a half-way reference point [26] and number familiarity [8]. Ebersbach et al. [8] posited a model composed of two linear segments, with the change point an indicator of number familiarity. Thompson and Opfer [17] found that the segmented model change point varied depending on number scale and questioned the claims of Ebersbach et al. [8]. Importantly, Opfer, Siegler and Young [27] still maintain the validity of the transition from logarithmic to linear representations, and caution that the fit of power models, as used by Barth and Paladino [26], could be influenced by the noise created during averaging procedures. Using an eye-tracking methodology, along with gaze pattern and fixation analysis Schneider et al. [28] found that children in the first years of school do spontaneously use orientation points (external markers) to support spatial-numerical processes such as numerical estimation. In light of these studies ([17,27,28]) we chose to focus on the established logarithmic and linear (not segmented) models and have only included the power model at the coefficient of determination analysis, with subsequent analyses focusing on individual target digits which were likely to have been positioned with the aid of an external factor or strategy.

A common strategy would be to utilize the number line itself, providing two external anchor points that could improve estimation accuracy of numbers located within close proximity to the end numbers (e.g. 97 is 3 units back from 100). The youngest participants may be limited to a counting strategy and may not factor in the spatial representation, as is their level of conceptual understanding. A more advanced possibility, as posited by both Barth and Paladino [26] and Ebersbach et al. [8], could be the application of mental anchor points, however, such a strategy would require understanding of the part-whole (proportion) relation. For example on a scale of 0-100, 50 could be a mental anchor point that arises from the participant dividing the number scale into two equal parts and matching this spatial division with the knowledge that 50 is half of 100. As with external anchor points, estimations that occur near these mental partitions are likely to have increased accuracy in comparison to those numbers located a greater distance away from a reference point; as suggested earlier, further individual variation is likely to be associated with number knowledge. Studies ([29,30]) have given corrective feedback for target digits near a particular landmark (150 on a 0-1000 number line) and found that estimates were less accurate when the target numbers were more distant from the landmark. This evidence from feedback studies (e.g. [29,30]) indicates that children can utilize both external and mental anchor points. In the Barth and Paladino [26] study the midpoint was highlighted to participants prior to commencing the task, but this prompt was not an inclusion within the present investigation.

This study further examines the putative logarithmic-linear shift of mental representations for the familiar number range (0-20) with children in Years 1, 2 and 3; with a focus on determining if external factors (i.e. strategy) may be at play during the estimation of specific target digits. Using the most established logarithmic and linear regression analysis of Berteletti et al. [7], Booth and Siegler [2]; Siegler and Booth [5] and Siegler and Opfer [10] as a foundation, this research extends the analysis systematically to investigate estimation behaviors of individual target numbers. First, a logarithmic or linear best fit model will be determined for individual data and used as a reference point to the Berteletti et al. [7] study that also utilized a number line with a maximum of 20. Second, the residuals to each target digit from both the logarithmic and linear models will be investigated to identify how well the two models represent the estimates of specific numeric values. Third and finally, the accuracy of estimation for individual target digits will be scrutinized, without any regression modeling. It is proposed that employing a familiar number range will increase the likelihood of strategy application and the novel analysis will indicate individual digits which might be the target of selective strategy use as suggested by Ebersbach et al. [8] and Thompson and Siegler [11]. It is hypothesized that the developmental shift from logarithmic to linear mental representation, after approximately two years of school ([2,5,10]), will also be the transition period where strategy use becomes evident. Based on existing speculation (e.g. [8,26]), this is likely to be located in close proximity to external anchor points or, in an advanced circumstance, mental anchor points which require the division of the number line into equal portions. For this reason, the present study is interested in main effects, but also the interaction between the variables, with a particular focus on separate year group behaviors. For example, it may be for particular target digits that the linear and logarithmic models demonstrate the greatest disparity, and that this varies developmentally. It is intended that this information will inform subsequent investigations that will seek to determine the various components (e.g. cognitive mechanisms, learned strategies) that contribute to the developmental path from logarithmic to linear representations and/or to the development of strategy use in numerical estimation, and any relationship between mental representations and strategy use.

## Methods

### Participants

Participants were 67 British children from Years 1, 2 and 3 (**Year 1: **n = 20, mean age 6.4 ± 0.24 years, 11 females. **Year 2: **n = 24, mean age 7.3 ± 0.33 years, 13 females. **Year 3: **n = 23, mean age 8.5 ± 0.36 years, 10 females). All participants performed within one and a half standard deviations of the mean on a brief version of the Wechsler Intelligence Scale for Children, 3^rd ^edition (WISC-III: Vocabulary, Blocks and Digit Span); with no significant difference between the year groups for the WISC-III triad (F (2, 64) = 1.592, p = 0.211).

Written informed consent was obtained from the parents/guardians of the children in this study. The study obtained ethical approval from the Cambridge Psychology Research Ethics Committee.

### Task and procedures

The assessment instrument was a number-to-position (NP) pencil and paper task, where participants were given a number and had to mark its position on a 160 mm number line. The number line had the range of 0-20 and the target digits were evenly distributed across the scale with 2, 4, 7, 8, 11, 13, 16 and 17. Two examples (3 and 9) on a 0-10 scale were completed together by the participant and researcher prior to commencement; this was discussion based to ensure understanding of the task and did not propose any strategies. During the testing trials (0-20) no corrective feedback was provided, just encouragement to continue onto the next item.

### Analysis

The NP task was assessed in the following way: The distance from the left end point (zero on the number scale), to the participant marking was measured in millimeters. The distances to the line markings were converted to numerical estimates for each target number. Mirroring the method employed by Siegler and colleagues [2,5,10], the following equation was used to determine the target number estimates:

$$\frac{Distance\;from\;left\;end\;point\;to\;marking\;\left(mm\right)}{Total\;length\;of\;line\;\left(mm\right)}\times scale\;of\;number\;line$$

An example of this calculation if a mark was placed 25 mm from the left end point of a 0-20 number line would be 25 ÷ 160 × 20; which means the target number estimate equates to 3.125.

The target number estimates were used in two main analyses; regression modeling and estimation accuracy for individual target digits. The regression modeling built on the method initially utilized by Siegler and Opfer [10] and two derived measures were used: the Coefficient of Determination values for individual participants, as well as Model Residuals for group level models. The unique component of the analysis was that of Estimation Accuracy. Taking the foundation from Siegler and Booth [5], this research gained a more detailed perspective into the numerical values that are likely to be estimated more accurately due to the contribution of external factors, such as learned strategy.

#### Coefficient of Determination

This analysis began with fitting linear and logarithmic models to the target number estimates, for each participant. Then for individual models (linear and logarithmic) a coefficient of determination (R^2^) was calculated. Comparing the linear coefficient (R_Lin_^2^) and logarithmic coefficient (R_Ln_^2^), for each child, it could be determined which model best represented each child's mental representation. The coefficient of determination values were entered in a 3 × 2 ANOVA. Factors were: Year (Year 1, 2 or 3) × Model (Linear or Logarithmic).

**Figure 1:**
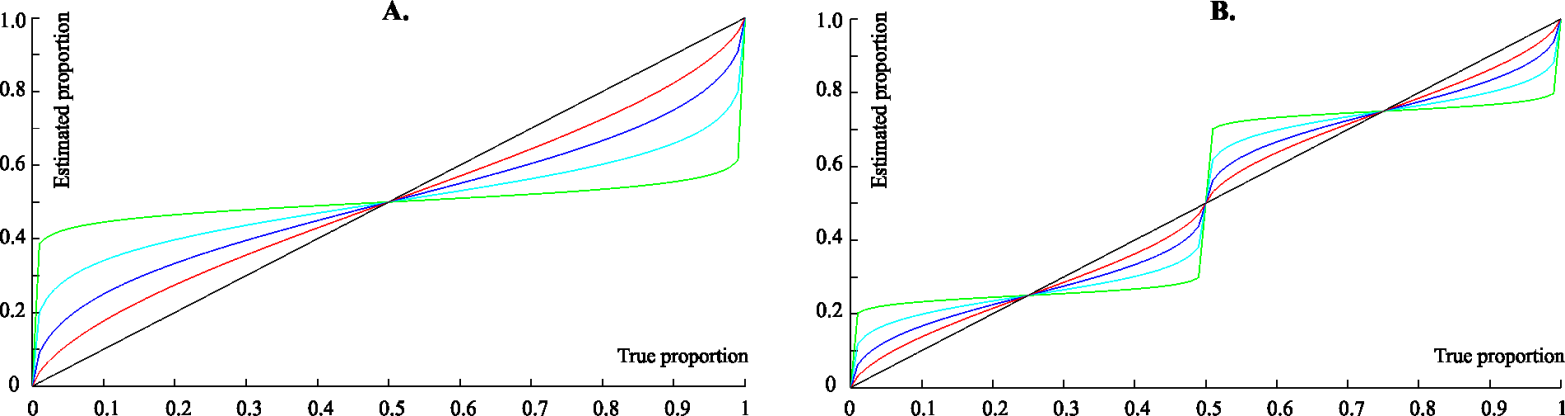
**Prediction proportion judgment cyclic power models as used in Hollands and Dyre **[**31**]**and Barth and Paladino **[**26**]. **a) **1-cycle model, with no central reference point; **b) **2-cycle model, with one central reference point. In both **a) **and **b) **β is the exponent in the power function describing the relationship of psychological to physical magnitude. Legend: Green β = 0.1, Aqua β = 0.3, Blue β = 0.5, Red β = 0.7, Black β = 1.0. NB. When β = 1, the function is linear.

In addition to this analysis, we explored the proportion judgment power model adopted by Barth and Paladino [26]. On an individual basis, for both 1-cycle and 2-cycle models, values of R^2 ^were determined, along with the parameter β (the exponent determining the shape of the power function relating psychological to physical magnitude). We selected the β with the highest R^2 ^and subsequently compared the best R^2 ^for the 1-cycle and 2 cycle models (Figure 1)[31]. These findings were interpreted in relation to the logarithmic-linear shift, with β = 1 corresponding to a linear model and then the further the value from 1, the closer to a logarithmic model. This additional model was then entered into a separate 3 × 3 ANOVA. Factors were: Year (Year 1, 2 or 3) × Model (Linear, Logarithmic or Power).

#### Model Residuals

Following the approach of Siegler and Opfer [10] the target number estimates were tabulated and analyzed at a group level. The median score for each year group was used to generate a graph of estimates versus actual target numbers. The median was selected because it is less affected by outliers, which could occur in this type of task. Each year group graph was then used to calculate both a linear and logarithmic regression model. The Siegler and Opfer [10] method calculated residuals to the group level median values and entered this into a paired-samples t-test. This would only determine if there significant difference between the residuals of the two models overall, omitting the variation of model residuals that could occur for individual target numbers. Extending the approach of Siegler and Opfer [10], the present study calculated residuals to individual target number estimates of each participant. This allowed the residuals to be entered into a more powerful 3 × 8 × 2 repeated measures ANOVA. Factors were: Year group (Year 1, 2 or 3) × Target Number (2, 4, 7, 8, 11, 13, 16 or 17) × Model (Linear or Logarithmic). This analysis allowed for a more detailed examination of how the model residuals varied for each target number and whether this interacted with year group.

To add further descriptive detail we followed the approach of Geary, Hoard, Byrd-Craven, Nugent and Numtee [32] and used the absolute residuals (from the group level models) to classify all trials as linear or logarithmic based on whether the child's estimate was closer to the predicted value of the linear of logarithmic model (i.e. which model produced the smaller residual). When the residuals of the linear and logarithmic models had a difference less than ± 0.4 units these trials were classified as ambiguous; this value was determined based on the distribution of the individual residuals.

#### Estimation Accuracy

The absolute percent error for each target number was calculated according to the equation:

$$\left|\frac{Estimate\;-\;Target\;Number}{Scale\;of\;number\;line}\right|\times 100$$

If the estimate was determined to be 3.125 and the target number 2, the equation would be |(3.125-2) ÷ 20| × 100 to obtain the error of 5.625%. In an attempt to reveal information about estimation accuracy of individual target numbers, and thus the potential application of external and mental anchor point strategies this study extends the method to reveal accuracy details about individual target numbers. This more detailed information was maintained and the percent absolute error values were entered into a 3 × 8 ANOVA, with factors: Year group (Year 1, 2 or 3) × Target Number (2, 4, 7, 8, 11, 13, 16 or 17). The Greenhouse-Geisser epsilon (ε) correction for sphericity was used in all ANOVAs whenever necessary. Reporting indicates the original degrees of freedom, the epsilon value, followed by the corrected (more conservative) significance level. All post hoc analyses were Tukey HSD tests. In the results section values represent mean ± standard deviation, unless otherwise stated.

To further investigate the potential for external and mental anchor points, we sought to explore the standard deviation of estimates as a function of target number, used by Cohen and Blanc-Goldhammer [33]. This was conducted on a year group basis in conjunction with the percent absolute error; lower standard deviations could pinpoint the location of an external or mental anchor point that was consistently applied by members of a year group.

## Results

### Coefficient of Determination

50% of Year 1, 75% of Year 2 and 74% of Year 3 children had higher coefficients of determination for the linear rather than the logarithmic models. The analysis of the coefficient of determination values revealed that the main effect of Model was significant (F (1, 64) = 9.96, p = 0.002, η^2 ^= 0.129). That is, the linear model (R^2^_Lin _= 0.87 ± 0.20) explained a greater degree of variance than did the logarithmic model (R^2^_Ln _= 0.84 ± 0.18). There was a main effect of Year (F (2, 64) = 10.63, p < 0.001, η^2 ^= 0.249) because the amount of variance explained by either model was significantly lower for Year 1, than Year 2 (p < 0.001) and Year 3 (p < 0.001); there was no significant difference between Years 2 and 3. There was no Model × Year interaction.

As the power model is sensitive to noise [27], some participants were discarded from the analysis leaving 51 in total. In terms of 1- or 2-cycle models majority of the participants were best represented by a 1-cycle model, with β values often very close to 1 (Table 1). Table 1 presents the individual power model results in comparison to the coefficient of determination values (R^2^) of the best fit logarithmic and linear models reported in the previous paragraph. The separate 3 × 3 ANOVA returned similar results. There was a main effect of model and a main effect of year (Model: F (2, 96) = 11.92, ε = 0.83, p < 0.001, η^2 ^= 0.196; Year: F (2, 96) = 5.19, p = 0.009, η^2 ^= 0.177). Overall, the linear model (R^2^_Lin _= 0.90 ± 0.13) explained more variance than both the power (R^2^_Pwr _= 0.86 ± 0.15, p < 0.001) and logarithmic models (R^2^_Ln _= 0.88 ± 0.11, p = 0.007). The main effect of year also indicated that the variance explained by any of the three models was significantly lower for Year 1, than Year 2 (p = 0.01) and Year 3 (p = 0.02); there was no significant difference between Years 2 and 3. There was no Model × Year interaction.

**Table 1:** Fits of power, logarithmic and linear models for individual children in Years 1-3

Year 1 *n = 13*					Year 2 *n = 19*					Year 3 *n = 19*				
**Power**			**Log**	**Linear**	**Power**			**Log**	**Linear**	**Power**			**Log**	**Linear**

**1-cycle or 2-cycle**	**β**	**R^2^**	**R^2^**	**R^2^**	**1-cycle or 2-cycle**	**β**	**R^2^**	**R^2^**	**R^2^**	**1-cycle or 2-cycle**	**β**	**R^2^**	**R^2^**	**R^2^**

1	0.78	*0.70*	*0.85*	*0.78*	2	0.53	*0.69*	*0.85*	*0.94*	1	1	*0.88*	*0.98*	*0.97*
1	0.7	*0.62*	*0.71*	*0.77*	1	0.73	*0.73*	*0.85*	*0.79*	1	0.82	*0.88*	*0.89*	*0.88*
1	0.84	*0.91*	*0.93*	*0.95*	1	1	*0.98*	*0.90*	*0.98*	1	1	*0.98*	*0.94*	*0.99*
1	0.88	*0.71*	*0.74*	*0.73*	2	0.81	*0.96*	*0.89*	*0.98*	1	0.88	*0.63*	*0.68*	*0.79*
1	1	*0.85*	*0.75*	*0.86*	1	0.97	*0.93*	*0.96*	*0.93*	1	1	*0.99*	*0.95*	*0.99*
1	0.92	*0.95*	*0.89*	*0.97*	1	0.89	*0.84*	*0.87*	*0.93*	1	1	*0.92*	*0.92*	*0.98*
2	0.75	*0.89*	*0.92*	*0.92*	2	0.75	*0.89*	*0.88*	*0.97*	2	0.8	*0.91*	*0.93*	*0.98*
1	0.47	*0.24*	*0.28*	*0.22*	1	0.74	*0.96*	*0.92*	*0.99*	1	0.67	*0.43*	*0.76*	*0.62*
1	1	*0.75*	*0.77*	*0.87*	2	0.93	*0.98*	*0.91*	*0.99*	1	0.93	*0.79*	*0.89*	*0.86*
1	0.73	*0.74*	*0.79*	*0.73*	1	0.69	*0.80*	*0.89*	*0.79*	1	0.98	*0.97*	*0.96*	*0.97*
1	1	*0.74*	*0.77*	*0.77*	1	1	*0.94*	*0.89*	*0.98*	1	0.83	*0.81*	*0.92*	*0.98*
2	0.69	*0.96*	*0.94*	*0.97*	1	1	*0.91*	*0.88*	*0.94*	1	0.76	*0.80*	*0.85*	*0.84*
1	1	*0.93*	*0.96*	*0.95*	1	1	*0.98*	*0.91*	*0.99*	1	1	*0.99*	*0.94*	*0.99*
					2	0.53	*0.87*	*0.91*	*0.95*	2	0.65	*0.96*	*0.86*	*0.96*
					1	0.95	*0.81*	*0.92*	*0.99*	1	1	*0.97*	*0.96*	*0.98*
					2	0.96	*0.97*	*0.94*	*0.99*	1	0.84	*0.87*	*0.97*	*0.99*
					1	1	*0.95*	*0.94*	*0.98*	1	1	*0.93*	*0.94*	*0.95*
					1	1	*0.97*	*0.87*	*0.90*	1	0.96	*0.99*	*0.94*	*0.99*
					1	0.93	*0.88*	*0.95*	*0.90*	1	1	*0.96*	*0.92*	*0.97*

For the power model it indicates whether a 1- or 2-cycle model was better supported by the data and the corresponding coefficient of determination (R^2^) and shape of the function (β). For logarithmic and linear models best supported by individual data, the R^2 ^values are reported.

Using individual data and individual regression models, these results indicate that in Years 2 and 3 the majority of participants had higher R^2 ^values derived from a linear model, indicating a linear best fit model. This was not the case with Year 1 as the variance explained, by any model, was significantly lower. This information supports our hypothesis that Year 2 indicates the onset of the dominance of the linear mental representation. Importantly, these findings do not clearly argue for a logarithmic to linear shift, as that would require the Year 1 participants to have the highest variance explained for a logarithmic model; what these findings argue for the dominance of a linear representation in Years 2 and 3 only.

### Model Residuals

**Figure 2:**
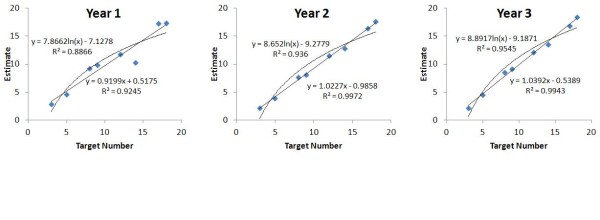
Median estimates for each target number, with linear and logarithmic regression equations for Years 1-3

This approach utilized individual residuals for each target number, calculated from group level median models (Figure 2). The linear model had a significantly smaller mean residual than did the logarithmic model (F (1, 51) = 36.71, p < 0.001, η^2 ^= 0.388. Linear vs. Logarithmic: 1.72 ± 1.96 and 2.01 ± 1.78 units, respectively). The Model × Year interaction was significant (F (2, 51) = 3.43, p = 0.040, η^2 ^= 0.073). Linear model residuals were smaller than logarithmic model residuals for Years 2 and 3, but not Year 1. For Year 1 participants, the mean linear residual (2.45 ± 2.33 units) was found to be not significantly smaller than the mean logarithmic residual (2.56 ± 2.18 units). The main effect of Year was marginal (F (2, 51) = 3.00, p = 0.058, η^2 ^= 0.105).

Overall the interaction of Target Number × Model was significant (F (7, 357) = 8.77, ε = 0.55, p < 0.001, η^2 ^= 0.143). Post hoc comparisons indicated that linear residuals were significantly smaller than logarithmic for target numbers 2 (p < 0.001) with a residual difference of 1.1 units, and 17 (p < 0.001) with a 0.7 unit difference in residuals. This difference was not significant for any other target numbers. These numbers were the focus of separate year group analyses (Table 2), with significant effects in Years 2 and 3 only.

**Table 2:** Statistical results for separate year group analyses for the Target Number × Model interaction (including post hoc analyses for target numbers 2 and 17)

		Residuals from group level models (mean ± SD)				
		
Group	Interaction Target Number × Model	*Model*	*Tukey HSD*			
			*Target number 2*		*Target number 17*	

**Year 1**	F (7, 91) = 2.02, ε = 0.54, p = 0.06, η^2 ^= 0.134	*Linear*	0.9 ± 0.7	*ns*	2.1 ± 2.2	*ns*
		*Logarithmic*	1.5 ± 1.1		2.6 ± 1.8	

**Year 2**	F (7, 133) = 5.30, ε = 0.45, p < 0.001, η^2 ^= 0.218	*Linear*	0.5 ± 0.7	*p < 0.001*	1.8 ± 2.4	*p = 0.09*
		*Logarithmic*	2.1 ± 1.7		2.5 ± 1.9	

**Year 3**	F (7, 133) = 3.59, ε = 0.43, p = 0.001, η^2 ^= 0.159	*Linear*	0.7 ± 0.4	*p < 0.001*	1.0 ± 0.9	*p = 0.001*
		*Logarithmic*	1.8 ± 0.7		1.9 ± 0.6	

Using the approach of Geary at al. [32] we further explored how model residuals would be distributed if we included an ambiguous category in addition to linear and logarithmic. As described in the methods, an ambiguous trial would occur when the residuals of the linear and logarithmic models had a difference less than ± 0.4 units. The overall percentages of trials classified as linear, logarithmic or ambiguous are provided in Table 3, including a breakdown by individual target digits in Table 4. These classifications support the residual Model × Year analysis, with Years 2 and 3 demonstrating a higher percentage of trials classified as linear in contrast to logarithmic, and the linear dominance less clear in Year 1. Overall, this analysis of model residuals has further explored the linear and logarithmic mental representations and the transition at Year 2, but this time highlighting specific target digits in relation to a group level model.

**Table 3:** Analysis of model residuals, by year group

Group	Residual fit (trial-by-trial)			Residual from group level models			
	
	*Linear*	*Logarithmic*	*Ambiguous*	*Linear*		*Logarithmic*	
	
	*%*	*%*	*%*	*M*	*SD*	*M*	*SD*
**Year 1**	46	37	17	2.5	2.3	2.6	2.2
**Year 2**	56	25	19	1.5	1.8	2.0	1.6
**Year 3**	54	21	25	1.3	1.6	1.7	1.4

Percentage of trials with best residual fit for linear and logarithmic models, or ambiguous (both linear and logarithmic model residuals within ± 0.4 units of one another). The overall mean ± standard deviation of the model residuals are summarized by year group.

**Table 4:** Percentage of trials with best residual fit for linear and logarithmic models, or ambiguous, by year group and target number

**Group**	**2**			**4**			**7**			**8**			**11**			**13**			**16**			**17**		
								
	***Lin***	***Ln***	***Amb***	***Lin***	***Ln***	***Amb***	***Lin***	***Ln***	***Amb***	***Lin***	***Ln***	***Amb***	***Lin***	***Ln***	***Amb***	***Lin***	***Ln***	***Amb***	***Lin***	***Ln***	***Amb***	***Lin***	***Ln***	***Amb***
							
***Year 1***	55	25	20	68	32	0	25	69	5	33	61	6	56	44	0	0	0	100	67	27	6	61	39	0
***Year 2***	96	0	4	63	29	8	57	39	4	60	36	4	50	37	13	0	0	100	63	29	8	65	26	9
***Year 3***	70	0	30	74	26	0	43	39	17	57	29	14	52	22	26	0	0	100	50	36	14	83	17	0

The ambiguous category was used when both the linear and logarithmic residuals were within ± 0.4 units of one another. Please note, that for target number 13, all are ambiguous because the residuals are identical as this is the location where the linear and logarithmic models intersect.

### Estimation Accuracy

There was a main effect of Target Number (F (7, 357) = 6.61, ε = 0.46, p < 0.001, η^2 ^= 0.109). Tukey post hoc comparisons indicated that the significant differences were in reference to the target numbers 2 and 4, which produced the lowest absolute errors. Error for target number 2 was significantly lower than for numbers 7, 8 and 13 (p < 0.01), and errors for target number 4 were significantly lower than for digits 7 and 13 (ps < 0.05). The mean estimation error rates decreased as years of education increased (**Year 1: **12.40 ± 7.51%, **Year 2: **8.39 ± 6.55%, **Year 3: **6.47 ± 3.91%), but the main effect of Year did not reach significance (F (2, 51) = 2.66, p = 0.079, η^2 ^= 0.095).

**Figure 3:**
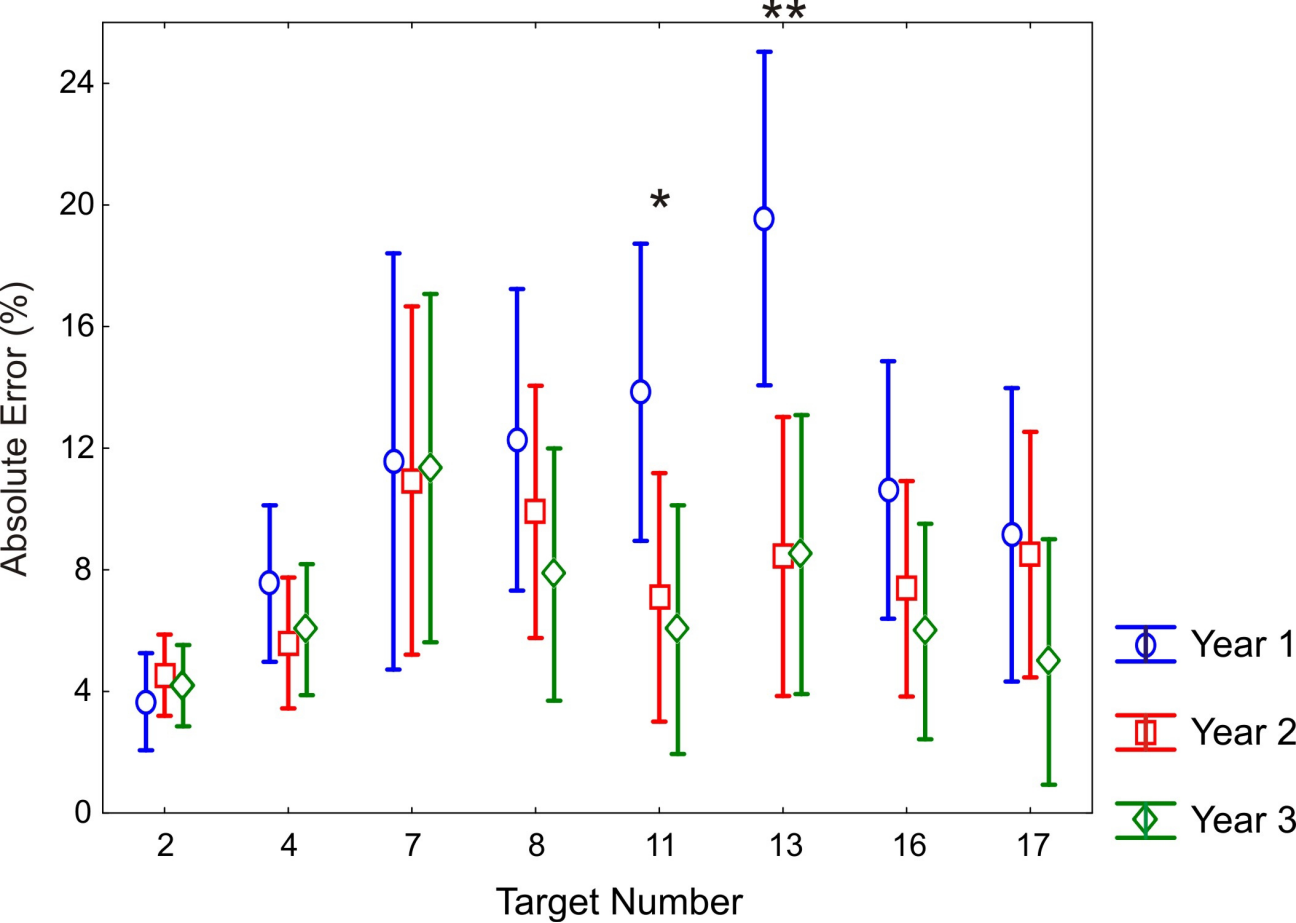
**The absolute percent error for each target number, by year group. **The error bars represent ± 95% confidence interval from the mean.

Figure 3 shows the marginally significant Target Number × Year interaction (F (14, 357) = 1.56, p = 0.087, η^2 ^= 0.051). To increase the confidence of this marginal finding and protect against a potential type 2 error, univariate analyses were completed and indicated that there were significant differences between the year groups for target digits 11 and 13 (F (2, 53) = 3.38, p = 0.042, η^2 ^= 0.117 and F (2, 53) = 6.10, p = 0.004, η^2 ^= 0.193, respectively). Closer scrutiny demonstrated that for target digit 11 the Year 1 mean error was higher and marginally significant in comparison to Year 2 (p = 0.09) and significantly higher when compared to Year 3 (p = 0.01). A similar pattern was evident with target digit 13; Year 1 children had a mean error which was significantly higher than both Years 2 and 3 (p < 0.01). Years 2 and 3 produced no significant differences for target digits 11 and 13.

**Figure 4:**
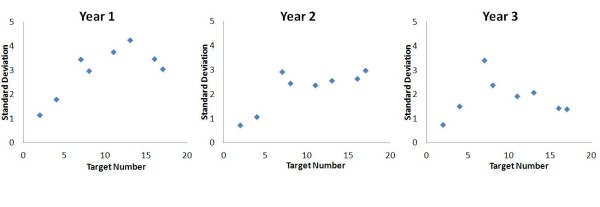
The standard deviation of estimates for individual target numbers, by year group

Separate Year group ANOVAs indicated a main effect of target number for Year 1 children (F (7, 91) = 4.33, p = 0.006, η^2 ^= 0.250). Post hoc analysis of Year 1 data again pointed towards target digits 11 and 13 and this is evident in Figure 3. The accuracy of estimating 13 was significantly poorer than the estimation accuracy of numbers 2, 4 and 17 (ps < 0.05). This significant accuracy difference was also evident with positioning target numbers 11 and 2 (p < 0.001). In contrast to the Year 1 children, there was no effect of target number in Years 2 and 3. Overall, this analysis has examined number line estimation, without regression modeling, in order to pinpoint individual digits that might be the target of selective strategy use. The year group comparisons indicated that digits 11 and 13 were poorly estimated by the Year 1 participants, thus separating them from Years 2 and 3. This was further confirmed through the comparison of the standard deviation of estimates for each target digit, with 11 and 13 with the highest standard deviation in Year 1 (Figure 4).

## Discussion

The aim of this investigation was to explore the developmental transitions of the mental representations associated with numerical estimation (logarithmic-linear shift). This was achieved by focusing on the estimation behaviors of individual target digits within a familiar number range (0-20), adapting and extending the methods of Siegler and colleagues [2,5,10] and building on the ideas of Barth and Paladino [26], Berteletti et al. [7], Ebersbach et al. [8] and Thompson and Siegler [11]. The statistical analysis of individual numbers inferred that there is merit in future in depth analyses of strategies application in conjunction with regression modeling.

On a developmental front, Year 1 children did not demonstrate a dominance of any representation in either the individual regression models or group level residuals. In fact, looking at the individual regression models alone, the variance explained by any model (linear, logarithmic or power), was significantly lower in Year 1 than in Years 2 and 3. When examining the group level model, children in Years 2 and 3 demonstrated the lowest residuals from a linear model, in comparison to a logarithmic model. The complexity of this transition to a linear mental representation is indicated by the categorization of residuals into linear, logarithmic and ambiguous (Table 4). For selected target digits, there were a high percentage of Year 1 participants who demonstrated linear-like behaviors however this was not always the case (e.g. target digits 7 and 8, Table 4). In contrast, Years 2 and 3 demonstrated a consistently high percentage of participants with trials that were best represented by a linear model. This matches the trend observed across the three groups of preschool children in the Berteletti et al. study [7]. In the familiar number scale tasks used in that study (1-10 and 1-20) the youngest group (mean age 4 years) did not demonstrate a bias towards either representation, meanwhile the middle and oldest groups (4.5-6.5 years) indicated a significantly lower linear residual. Furthermore, the present findings are in line with the 0-100 number line developmental findings of Siegler and colleagues [2,5,10] except in Year 1 with no significant bias.

Extending the analysis to include 1- and 2-cycle power functions [26], in this case, did not create any further clarity in terms of R^2 ^values. Perhaps it was the reduced number range (0-20) and minor differences in task instructions that limited the potential for power models to represent the data, as Barth and Paladino [26] focused on a 0-100 number line and indicated 50 as being 'halfway' at the beginning of the experiment. The individual data in the present study was typically best fit by a 1-cycle model (Table 1), which aligns with the fact that participants were not directed towards the 'halfway' point when task instructions were given. Further exploration into the use of proportional power models is required, particularly in relation to the appropriateness of using such an approach, as highlighted by Opfer et al. [27].

Extending the work of Siegler and Opfer [10] the results from the individual target digits are a unique contribution to the body of literature, as this begins to explore the possibility that the development of mental representations could be marked by the use of the external 'anchor points' as described by Ebersbach et al. [8]. In the case of this research application of external anchor points should be observed for target digits 2 and 17. According to existing research [2,5,7,8,10], a low linear residual would indicate a more accurate mental representation. The number scale 0-20 is familiar to students in Years 1-3, however, the strategies involved in positioning numbers accurately on an externally represented number line may not be fully established. For Year 1 the linear and logarithmic residuals for both of these numbers were similar (Table 2). Year 2, showed a significantly lower linear residual for target number 2, and perhaps as a function of the magnitude effect or incomplete transition, only a marginal preference for a linear model for target number 17 (Table 2). Finally then, for Year 3, the difference between linear and logarithmic residuals for target numbers 2 and 17 was significant with a linear model providing the lowest residual (Table 2). This indicates a developmental transition, but also highlights that the greatest disparity between the logarithmic and linear models is likely to occur in close proximity to external anchor points. It is this external anchor point reasoning that we use to speculate that both target digits 2 and 17 have lower linear residuals in comparison the logarithmic model residuals.

Parallels can be drawn between the observations of the present study and the developmental progression seen with arithmetic strategies in the classroom. Existing research (e.g. [34-36]) purports the importance of sequences and counting in the early stages of development, but also identifies that the application of the base-ten structure in constructing novel relationships among numbers up until approximately 9 years of age. As a further example, Dutch mathematics education programs teach mental arithmetic strategies that employs decomposition as the basis of instruction. From Grade 2, Dutch children are encouraged to use mental jumps and decomposition, often beginning on a number line, in order to encourage flexible mental strategies [37]. Given this information and linking back to the present data, it is proposed that the Year 1 children did not demonstrate any clear anchor point application because they were limited to counting strategies and were unable to link the numerical value to the spatial cues provided by the number line. Subsequently, in Years 2 and 3 we do see evidence of the continued development of more flexible strategies, and use of anchor points, that utilize decomposition and part-whole relations.

A question that comes out of this discussion is, given the flexibility of strategy application, is it in fact meaningful to try and model the mental representation of numbers using a fixed linear/logarithmic model? What the previous paragraph has posited is that specific numbers could exhibit unique behaviors as a function of the familiarity with the number range, proximity to either external or mental anchor points, as well as knowledge of arithmetic strategy. While Ebersbach et al. [8] focused on the role of external anchor points, the mental anchor points in particular would relate to more advanced strategy application, such as knowledge of proportions (e.g. half, quarter etc.) and ability to mentally partition the external number. This potential for individual difference represents a limitation of the linear/logarithmic modeling approach. In the followings we discuss what a more detailed approach could add to the current knowledge base.

Siegler and Booth [5], in their first experiment with 0-100 scale, had error rates of 27% for Kindergartners, 18% for Grade 1 and 15% for Grade 2. The later Booth and Siegler [2] study demonstrated a more obvious plateau with Kindergartners: 24%, Grade 1: 12%, Grade 2: 10%, and Grade 3: 9%. This could be a function of the 0-100 number scale being in the unfamiliar range. In these two studies [2,5], the youngest groups were always significantly different to the subsequent year levels; however, this was not the case in the present research, as average percent absolute error was lower and there was no significant group difference.

Overall, the results from the percent absolute error data indicated that the most accurately estimated numbers on the 0-20 number line were digits 2 and 4. This could be for two reasons; first, referencing the lower values (2 and 4) to the external lower anchor point of zero or their knowledge of one. Second, it could be that number magnitudes up to 4 have a stronger representation, because these quantities are understood from infancy [38-40] in what Feigenson et al. [15] describes as the small number system. This could also be linked to how frequently these numbers are encountered in a young child's everyday language (e.g. [13,25]). This also fits with theories of enumeration and subitizing abilities being present prior to verbal counting (e.g. [41-43]). The fact that these enumeration and subitizing abilities are limited to numerosities of four (e.g. [44,45]), and that they are present from infancy, could explain the strength of the representation of digits 2 and 4, and thus producing more accurate estimations. This is further evidenced in the categorization of trials (Table 4), where all year groups demonstrated a high percentage of linear classifications of digits 2 and 4. Then for the subsequent numbers 7 and 8, Year 1 stood out with a high percentage of logarithmic classifications, which were not evident in Years 2 and 3 (Table 4). It is our interpretation that for Year 1 digits 7 and 8 are likely to be less frequently encountered and could contribute to the variation.

On the developmental front, the Year 1 children again showed separation from the older year groups in the estimation accuracy of individual target digits. For the positioning of numbers 11 and 13, Year 1 children produced estimates that were significantly less accurate than Years 2 and 3 (Figure 3). This gained further support when investigating the standard deviations for each target digit, with the greatest variation evident for digits 11 and 13 for Year 1 participants (Figure 4). The idea of applying external anchor points to aid in the estimation of numbers has been purported, although only briefly in existing studies [5,8]. However, it could be argued that a further number estimation strategy could be to apply mental anchor points and divide/partition the external number line into segments which would increase the accuracy of positioning. The Year 2 and 3 children of this study were probably exhibiting these behaviors. A potential mental partition would be that of halfway, and number 11 was the closest target digit to the mental anchor point of 10. It is proposed that, in Year 1, as both a result of both mental representation (no clear logarithmic-linear preference) and educational experience, children lacked the requisite representations/skills to apply mental anchor point strategies and accurately estimate these central numbers (i.e. 11 and 13). The core learning concepts for Year 1, as prescribed by the National Framework in England focus on counting and skills related to addition and subtraction, not division or the part-whole relation. The early understanding of division principles often stems from the relationships between doubling and halving, which is promoted in Year 2. This knowledge of 'half' is required for the application of mental anchor point strategies, which as a function of educational experience the Year 1 children do not typically possess. The fact that the formal teaching of this concept in Year 2, coincides with increased accuracy of estimation for central digits (Figure 3), lends further support to this argument. Further detailed analyses would be required to strengthen these proposals and will be the focus of subsequent studies.

Examining percent absolute error for specific target numbers allowed the discussion to go beyond the limitations of structured modeling to further explore the potential of strategy application. The merits of both external and mental anchor points, as estimation strategies were supported. In line with the initial hypothesis, Year 2 appeared to represent a transitional phase, with the apparent onset of part-whole strategies to aid the creation of mental anchor points. It was hypothesized that the developmental shift from logarithmic to linear mental representation would likely coincide with evidence of strategy use; the present study supports this.

The development of the anchor point strategy application could be described as follows; the first would be to utilize the external anchor points to assist positioning of the numbers. Children in the first year of school may only use the left most point, rather than having the strategic knowledge to employ both extremes. The developmental progression, along with the logarithmic-linear shift, extends to include the use of both anchor points after Year 1. This is followed by some level of mental partitioning, which again advances in complexity and is the result of educational experience, which became evident in Year 2 (Figure 3). It would be an interesting investigation to more closely map the development of these individual strategies and educational experience with the logarithmic and linear modeling scenarios. This extension would ideally include saturation of all target digits within the prescribed number range and a direct means of determining the strategy used to solve the problem, whether requesting verbal reports from participants or applying the use of eye-tracking technology as introduced by Schneider et al. [28]. Furthermore, it would be worthwhile to investigate whether the type and flexibility of strategies used during these first years of school could predict later mathematical achievement. It is the combination of these proposals that could facilitate the most meaningful insights for informing education.

## Conclusions

To summarize, prior cognitive research into children's numerical estimation behaviors has argued for both logarithmic and linear models being able to describe the mental representation; with different models having dominance depending on development and familiarity of the number range. To convert this understanding to being more applicable for educational practice and understand the path from logarithmic to linear dominance the present study took this theoretical basis and conducted a number-to-position number line estimation task with children from Years 1-3. The use of a familiar (0-20) number line meant that our analysis could extend beyond the more abstract linear and logarithmic modeling interpretations and examine the variable strategic behaviors associated with individual target numbers. This analysis provided the most meaningful link to strategy application and identifies a future direction of research. Results indicated that when operating within a familiar number range, a linear representation dominates from Year 2, but also there was indication that beginning in Year 2 children start to apply estimation strategies, which become more advanced in Year 3. These most advanced children provided evidence of applying external anchor point strategies for lower and upper bound target digits, as well as the possibility of mental anchor points. For Year 1 children, positioning central numbers such as 11 and 13 seemed to produce the highest errors, whereas children in Years 2 and 3 were more accurate and were thought to be applying a 'halfway' mental anchor point that improved the placement of central numbers. This study concludes that further scrutiny of estimation strategies, when combined with modeling techniques, could greatly increase the understanding of developing mental representations.

## Competing interests

The authors declare that they have no competing interests.

## Authors' contributions

Both SW and DS took part in planning and designing the experiment. SW completed the data collection and analyses and drafted the manuscript. DS assisted in preparing the manuscript. All authors read and approved the final manuscript.
